# Ligamentous injury-induced ankle instability causing posttraumatic osteoarthritis in a mouse model

**DOI:** 10.1186/s12891-022-05164-5

**Published:** 2022-03-08

**Authors:** Junkun Li, Zhi Chen, Yu Cheng, Chao Gao, Jiaxin Li, Xiaohui Gu, Fan He, Zongping Luo, Huilin Yang, Hongtao Zhang, Jia Yu

**Affiliations:** 1 Department of Orthopedics, The First Affiliated Hospital of Soochow University, No.188 Shizi St, Suzhou, 215006 Jiangsu Province P. R. China; 2 Orthopedic Institute, Medical College, Soochow University, No.708 Renmin Rd, Suzhou, 215006 Jiangsu Province P. R. China; 3 Department of Orthopedics, Fengcheng Hospital of Fengxian District, Shanghai, P. R. China; 4 Department of Orthopedics, Changshu Hospital affiliated to Soochow University, Changshu No.1 People’s Hospital, No.1 Shuyuan St, Changshu, 215500 Jiangsu Province P. R. China; 5 Department of Data Science, Faculty of Mathematics, University of Waterloo, 200 University Ave W, Waterloo, ON N2L 3G1 Canada

**Keywords:** Ligamentous injury, Ankle sprain, Animal model, Footprint analysis

## Abstract

**Background:**

This study aims to explore the relationship between surgically-induced ankle instability and posttraumatic osteoarthritis (PTOA) in a mouse model, and to provide reference for clinical practice.

**Results:**

Ligamentectomy was performed on 24 eight-week-old male C57BL/6 J mice, which were divided into three groups. Both the anterior talofibular ligament (ATFL) and the calcaneofibular ligament (CFL) were severed in the CFL + ATFL group, while only the CFL was removed in the CFL group. The SHAM group was set as the blank control group. A wheel-running device was used to accelerate the development of ankle osteoarthritis (OA). Balance measurement, footprint analysis, and histological analysis were used to assess the degree of ankle instability and OA. According to the balance test results, the CFL + ATFL group demonstrated the highest number of slips and the longest crossing beam time at 8 weeks postoperatively. The results of gait analysis exhibited that the CFL + ATFL group had the most significant asymmetry in stride length, stance length, and foot base width compared to the CFL and SHAM groups. The OARSI score of the CFL + ATFL group (16.7 ± 2.18) was also much higher than those of the CFL group (5.1 ± 0.96) and the SHAM group (1.6 ± 1.14).

**Conclusion:**

Based on the mouse model, the findings indicate that severe ankle instability has nearly three times the chance to develop into ankle OA compared to moderate ankle instability.

**Supplementary Information:**

The online version contains supplementary material available at 10.1186/s12891-022-05164-5.

## Introduction

Ankle ligament sprain is one of the most common joint injuries in daily life and sport activities. An epidemiological study has revealed that the estimated incidence rate of ankle sprains in the general population presenting to emergency departments in the United States is 2.15 per 1000 person-years, and nearly half of all ankle sprains occur during athletic activity [38]. Up to 40% of patients with acute ankle sprain are likely to develop chronic ankle instability (CAI) [9]. CAI induces repeated impingement and friction of the cartilage surface, finally resulting into post-traumatic osteoarthritis (PTOA) [16, 17, 26, 32, 34]. So far, the pathogenesis of ligamentous injury-induced ankle PTOA still lacks convincing prospective research, and the specific treatment of ligamentous injury-induced PTOA still requires advanced recommendations.

Currently the main methods for investigating the biomechanics and pathophysiology of ankle PTOA include clinical retrospective comparison, finite element analysis (FEA), cadaveric studies, and gait analysis. Clinical researches on PTOA are basically based on retrospective studies that lack prospectiveness [15, 26, 34]. Previous basic studies on PTOA mainly include cadaveric experiments and FEA, but are not supported by in vivo experiments and lack authenticity. These two methods can not provide analysis from the perspective of cellular changes, and are limited by the sample size of the cadaveric experiments; thus, the level of evidence is relatively low [4, 25, 28]. Gait analysis of human motion has also been reported in a PTOA study [11]; however, no microscopic study of the cartilage was performed. Animal models for the research of PTOA have many advantages [31], such as knowledge of the exact time of the disease onset or of the initiating event, control of causes and environmental influences, consistent phenotype (expression of similar molecular responses to the same experimental intervention is expected), development of genetic models able to investigate the influence of specific genes and molecular mechanisms, and tissue samples collection at every stage of OA.

**Fig. 1:**
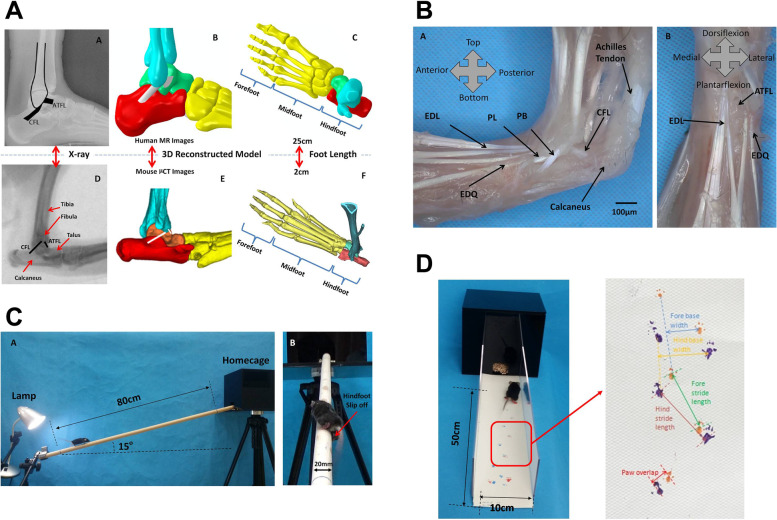
**A** Comparison of the anatomy similarity between human and mouse ankle using X-Ray and finite element analysis. **B** A schematic diagram of the surgical anatomy of the modeling process. **C** Schematic diagram of mouse balance beam project test. D Schematic diagram of mouse footprint analysis and test

Animal experiments not only can macroscopically observe behavioral gait changes, but also can analyze the ankle cartilage injury by histological methods. Previous studies on PTOA included mouse, rat, rabbit, pig, and horse as animal models. Considering the anatomical similarity and activity, mice or rats have been the most appropriate choice (Fig. 1A). A mouse model can be combined with surgery and dietary or exercise regimens to mimic human OA risk factors. Mice are reasonably inexpensive to house, and the progression of disease in these animals is quite fast [8]. A large number of assessment tools are now available for use in mice, such as micro-CT, gait analysis, and beam walking test. Several researchers have worked on ankle OA using rat/mouse models. Hubbard-Turner et al. used a surgical approach to cut off the different lateral collateral ligament to simulate different ankle sprains [19]. Chang et al. performed a histological study on ankle OA [7]. Further exploration based on previous studies, as well as cartilage damage assessment, behavioral tests, and injury level classification will be presented in the present study.

Currently, there are no known high-level evidence-based guidelines specific to the biomechanics and pathophysiology of PTOA. Thus, in the present study, ligamentous injury-induced PTOA, produced by surgically transecting the anterior talofibular ligament (ATFL) and the calcaneofibular ligament (CFL) of mouse hind foot, was macroscopically and microscopically investigated, in the aim of guiding clinical diagnosis and treatment. It should be noted that the animal model developed in this study was designed to investigate the mechanical ankle instability, not the functional ankle instability, and neurological factors were not taken into account.

## Methods

### Animal preparation

All experiments were performed in strict accordance with the guidelines for care and use of laboratory animals and were approved by the Animal Care Committee of Soochow University. Twenty-four specific pathogen-free C57BL/6 J male mice (8 weeks old; weighing 20–25 g) were purchased from a supplier. All experimental mice were divided into three groups with 8 mice in each group, *j.e.*, the ATFL+CFL group, the CFL group, and the SHAM group. All mice were maintained in our animal facility under specific pathogen-free conditions. All mice were housed in the University Vivarium with 12-h light/dark cycles during the whole course of the study under standardized room temperatures and relative humidity of 18–22 °C and 20–40%, respectively. The animals were houses in groups of four, fed with a standard diet, and had ad libitum access to water. Baseline measurements of gait and balance were conducted on all mice before being randomly allocated to a surgery group (SHAM, CFL, and ATFL+CFL group).

### Surgical procedures

Surgeons have been fully trained and certified in animal experiments. Each mouse was anesthetized with 4% isoflurane gas and supplemental oxygen. Then, the left ankle of all mice was shaved and cleaned with alcohol, followed by a Chlorhexidine scrub. After the site was prepared, each mouse remained under anesthesia and was moved to a sterile surgical field under a microscope. The techniques used by Kim et al. were employed to guide the transections. In the CFL group, a small incision was made under the microscope using sterile equipment [22]. The skin was retracted, the CFL was transected, and the skin was closed using 8–0 surgical sutures. In the ATFL+CFL group, after the skin was retracted, both the ATFL and the CFL were transected, and the skin was closed using 8–0 surgical sutures. In the SHAM group, a small incision was made in the same location as in the CFL and ATFL+CFL groups; however, no ligaments were injured and the skin was closed using 8–0 surgical sutures (Fig. 1B). After the operation was finished, the mouse was removed from anesthesia and placed in a recovery area. Each mouse received a subcutaneous injection of 200,000 U penicillin diluted with saline and was allowed to recover under a warming lamp until freely mobile. All mice were monitored for 24 h after surgery.

### Acceleration of ankle OA by forced wheel running

Each mouse was placed on a motorized running wheel equipment purchased from Shandong Yiyuan Technology Development Co. (Binzhou, Shandong, China) with a diameter of 20 cm and a width of 5 cm over an 8-week period according to the method of *Chang* et al. [7] The running wheel exposure consisted of placing each mouse on the wheel that was moving at a velocity of 10 m/min for 30 min. If the mouse got fatigued and failed to run, it would slide off the moving wheel on an electric shock grid with 1.2 mA of current and then it would have to keep running in order to avoid the electric shock. The electric shock time during wheel running could be recorded by the running wheel equipment.

### Balance measurement

The balance of the mice was assessed by measuring the ability of crossing an inclined (15°) narrow beam to reach an enclosed safety platform [37]. The beam was a 1-m-long round piece of wood with a diameter of 20 mm that was elevated above the bench surface and connected to an enclosed box (20 cm^3^) as a home-cage for the mouse to escape into [37]. The training of the mice consisted of placing them 10 cm away from one end of the beam and allowing them to cross the beam reaching the enclosed box. A mouse was considered to be trained if it was able to cross the beam in less than 20 s for three consecutive attempts. During test trials, mice were allowed up to 60 s to cross the beam. The duration to cross the beam and the number of times the left hind foot slipped off the beam were recorded as dependent variables (Fig. 1C).

### Footprint test

Footprints were collected by painting the hindfeet and forefeet of the mice with red and blue nontoxic paints, respectively. The baseline of each footprint was evaluated and corrected before footprint analysis. Mice was prompted to walk across a white sheet affixed to a 50-cm-long and 10-cm-wide passageway (with 10-cm-high walls) toward an enclosed cubic box (20 cm^3^), leaving a trace of their paw prints on the sheet. During each test session (3 days to 8 weeks after surgery), a fresh sheet of white paper was placed on the floor of the runway for each mouse (Fig. 1D). The dependent measures included stride length asymmetry, paw overlap asymmetry, hindfoot base width, forefoot base width, hindfoot stance length, and forefoot stance length [21, 37]. The stride length was defined as the average distance of forward movement between each stride using a heel-to-heel measuring technique. The stride length asymmetry was calculated as the average right-stride length divided by the average left-stride length. Thus, a value > 1 indicated a larger right-stride length and a value < 1 indicated a larger left-stride length. Paw overlap was used to measure the uniformity of step alternation. In order to quantify paw overlap, the distance between the center of the left and right fore and hind footprints was recorded. If the center of the hind footprint fell directly on top of the center of the preceding front footprint, a value of 0 was recorded. Asymmetry in left-to-right paw overlap was defined as the average right paw overlap distance divided by the average left paw overlap distance. Thus, a value > 1 indicated a larger right fore/hind foot overlap and a value < 1 indicated a larger left hind/forefoot overlap. The fore and hind foot width measurements were defined as the average distance between hind and front footprints, respectively. Width outcomes were defined as the perpendicular distance from the inside of the paws among the left and right steps of a mouse. Stance length outcomes were measured as the distance between forefoot and hindfoot [21]. For each gait outcome, the maximum number of values from each test trial was obtained, excluding footprints made while the mouse was initiating and terminating gait. The mean value of each set of outcomes was used for statistical analysis. A preliminary analysis of gait data was performed on 19 gait trials in order to establish both inter-and intra-reliability of measuring the selected gait outcomes. The intra-reliability, measured with intraclass correlation coefficient (ICC) values, was excellent for all outcomes (stride length = 0.99, paw overlap =0.92, hind foot width = 0.90, and forefoot width = 0.92). Similarly, the inter-reliability, also measured with ICC values, was excellent for three outcomes (stride length = 0.98, paw overlap = 0.92, and hind foot width = 0.90) and good for one outcome (forefoot width = 0.89). Finally, print selection is a concern when performing gait analysis in rodents [21]. Thus, the prints for analysis were independently selected by two investigators. The number of prints selected per gait trial demonstrated excellent inter-reliability (ICC = 0.96). Across 19 trials, the total number of prints that differed between the investigators was 9, less than half of a print per trial.

### Histological analysis

The mouse samples were fixed in 4% paraformaldehyde for 24 h, decalcified in ethylenediaminetetraacetic acid (EDTA) at 37°Con a shaker for 5 days, and embedded in paraffin. Frontal sections 6μmthick were prepared and stained with Safranin-O/Fast green according to a standard protocol. For the ankle tissue analysis, sagittal sections around the center of the talus were used. The talar articular cartilage (AC) of the tibiotalar joints was collected. The severity of the tibiotalar for ankle OA was quantified using the Osteoarthritis Research Society International (OARSI) scoring system [13]. Histological scoring was performed on tibial and talar AC of the tibiotalar joint using 5–10 slides for each sample.

### Statistical analysis

All data were obtained at the Orthopedic Institute of Soochow University. Statistical analysis was performed using the SPSS 22.0 statistical package. By referencing the previous study of *Turner* [19] and considering the sample size of our study, Two-way ANOVAs (group x time) were used to determine the significance of difference between the three groups included in the study. In this experiment, three comparisons were made: 1. Comparison between the CFL + ATFL and CFL groups; 2. Comparison between the CFL group and SHAM groups; 3. Comparison between the CFL + ATFL group and SHAM groups. Paired Student’s t-test was used for each group along the time axis to examine if the data has increased/decreased as the study processed. A *p*-value < 0.05 was considered statistically significant.

Prior to the trial, we evaluated the relevant elements of the randomized controlled study, including grouping method, blind method, intervention measure, main observation index. Cohen’s d was also calculated to evaluate the effect size between different groups in this experiment.

## Results

### Number of slips

**Fig. 2:**
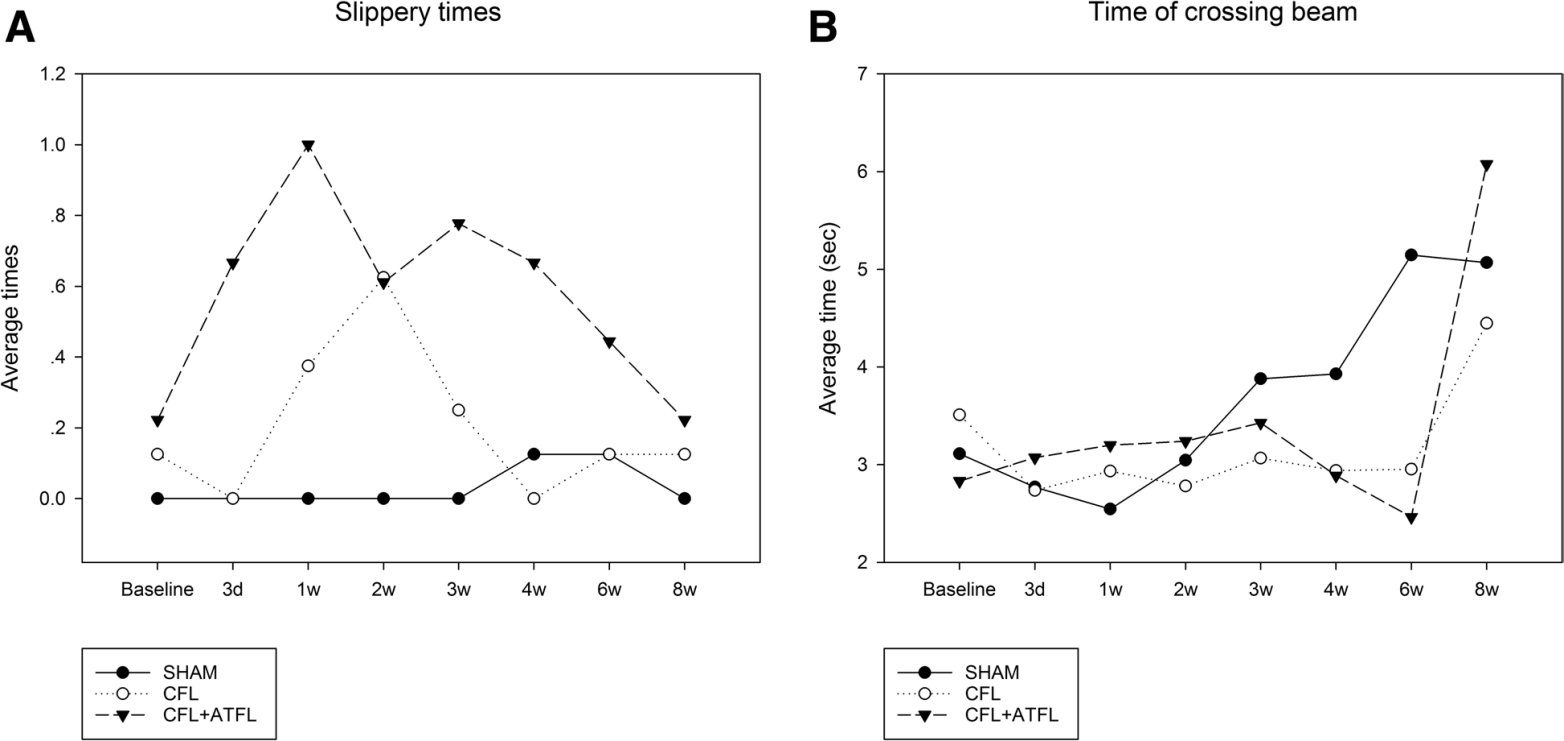
**A** Slippery times of different groups. **B** Time of crossing beam of different groups

Among the three tests, the one with the minimum number of slips was used for the calculations. Compared to the SHAM group, none of the groups demonstrated significant difference in baseline characteristics. Significant differences in Day3, Week1, and Week2 (*p* = 0.0275, 0.0207, 0.0003 < 0.05, respectively) were found between the CFL + ATFL group and the SHAM group. The Cohen’s d was 0.617. The CFL group demonstrated a significant difference (*p* = 0.0005 < 0.05) in W2, but no significant difference (*p* = 0.0787 < 0.1) was found in Week1. No significant difference (*p* > 0.05) was found between all groups and the SHAM group in W3. The *P*-values were computed by two-tailed t-test. When compared to its baseline data, the SHAM group showed no significant changes over 8 weeks. The CFL group demonstrated an increase in slips (*p* = 0.085238 < 0.1) on W1 and no significant increase or decrease was observed after that. The CFL + ATFL group showed a significant increase in Day3 (*p* = 0.005601 < 0.05), Week1 (*p* = 0.010469 < 0.05), Week3 (*p* = 0.039801 < 0.05), and Week4 (*p* = 0.024587 < 0.05) (Fig. 2A). The Cohen’s d was 0.881. *P*-values were computed by one-tailed paired t-test.

### Crossing beam duration

The average passing time is used for calculation. Both CFL and CFL + ATFL group used significant shorter time (*p* = 0.0312, 0.0110 < 0.05) than SHAM group on W6, there’s no other significant difference (*p* > 0.1).SHAM group showed significant decrease in the time consumed on D3 and 1 W when compared with baseline data (*p* = 0.015 < 0.05),while it showed significant increase on 3 W, 6 W, and 8 W. The Cohen’s d was 0.272. CFL group showed significant decrease on 2 W and 6 W and no significant change other than that. CFL + ATFL group showed significant increase on 8 W and no other significant change (Fig. 2B).

### Footprint analysis

#### Front stride length asymmetry

**Fig. 3:**
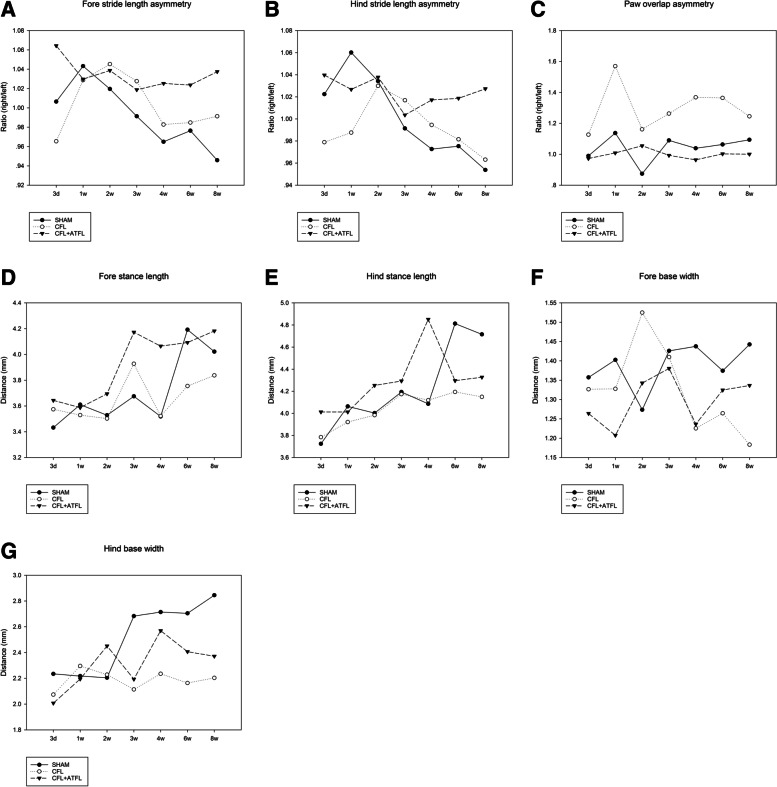
**A**, **B** Fore and hind stride length asymmetry of different groups. **C** Paw overlap asymmetry of different groups. **D**, **E** Fore and hind stance length of different groups. F, G. Fore and hind base width of different groups

No significant difference was found in front stride length asymmetry between the SHAM group and the CFL + ATFL group at 8 weeks after operation. At 2 weeks postoperatively, in the CFL group, the front stride length asymmetry was significantly increased compared to Day3 (*p* = 0.046 < 0.05) (Fig. 3A,B). The Cohen’s d was 0.118.

### Paw overlap asymmetry

Significant differences in paw overlap asymmetry between the CFL group and the SHAM group (*p* = 0.026 < 0.05), between the CFL + ATFL group and the SHAM group (*p* = 0.08 < 0.1),as well as between the CFL group and the CFL + ATFL group (*p* = 0.08 < 0.1) were observed. The Cohen’s d was 0.178. Moreover, there was a significant difference in the paw overlap asymmetry between the CFL + ATFL group and the SHAM group and the CFL group at the 8th week after operation (*p* = 0.0009, *p* = 0.002 < 0.05, respectively). The paw overlap asymmetry in the CFL + ATFL group at 8 weeks after operation was significantly higher than that at Day 3 (*p* = 0.0005 < 0.05) (Fig. 3C). The Cohen’s d was 0.549.

#### Hind foot width

The hind foot width of the CFL group was found to be significantly different from that of the SHAM group at the 3rd and 4th week after operation (*p* = 0.017, *p* = 0.011 < 0.05, respectively). At 2 weeks and 6 weeks postoperatively, no significant difference in hind foot width was found between the CFL group and the SHAM group (*p* = 0.06, *p* = 0.09 < 0.1, respectively). At Week 3, the CFL + ATFL group demonstrated significant differences in hindfoot width compared to the CFL group (*p* = 0.03 < 0.05). The Cohen’s d was 0.802. The hind foot width of the CFL group at 6 weeks after operation was significantly different from that at Day 3 (*p* = 0.025 < 0.05). Furthermore, there was a significant difference in hindfoot width between the 3rd, the 6th, and the 8th week after operation (*p* = 0.011, *p* = 0.018, *p* = 0.017 < 0.05, respectively) (Fig. 3F, G). The Cohen’s d was 0.331.

#### Hind foot stance length

Significant differences in hind foot stance length between the CFL group and the SHAM group were observed at the 4th week after operation (*p* = 0.041 < 0.05) and between the CFL group and the CFL + ATFL group at the 3rd week after operation (*p* = 0.043 < 0.05). The absolute distance between the CFL group and the CFL + ATFL group was significantly different compared to that of D3 (*p* < 0.05) at the 2nd, the 3rd, the 4th, the 6th,and the 8th week (Fig. 3D,E). The Cohen’s d was 0.161.

### Histological analysis

**Fig. 4:**
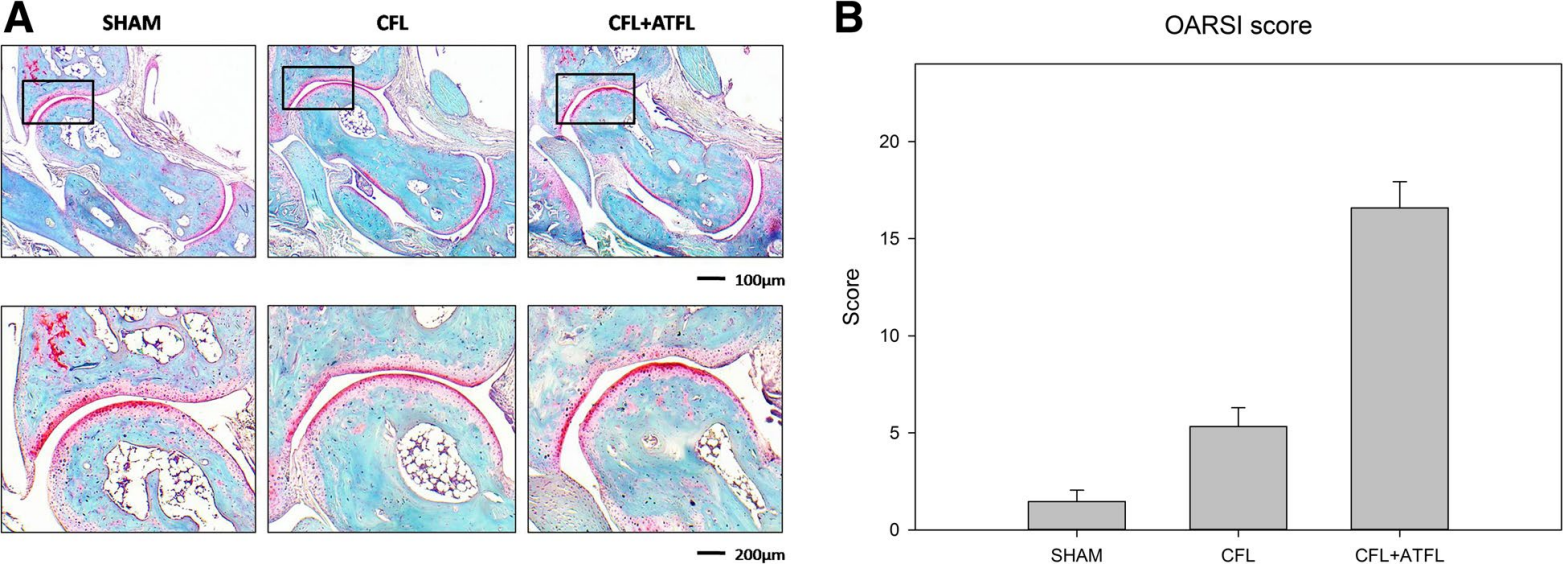
**A** Histology imaging of different groups. **B** OARSI score of different groups

Eight weeks after surgery, the ankle joint was significantly degenerated in the CFL + ATFL group compared to that in the SHAM group, while that of the CFL group was less changed. In the CFL + ATFL group, decrease in AC thickness and deficit at the edge of the AC were observed under the microscope (Fig. 4A). In the CFL group, the AC layer of the ankle joint was basically intact and only a few signs of joint degeneration were observed. The OARSI score for the CFL + ATFL group was 16.7 ± 2.18, for the CFL group 5.1 ± 0.96,and for the SHAM group 1.6 ± 1.14 (*p* = 0.032 < 0.05).After all sections were histologically graded, it was found that 73% of slices in the CFL + ATFL group met the criteria for OA, with only 25% in the CFL group, compared with SHAM group (Fig. 4B).

## Discussion

The feasibility of using mouse model to mimic human ankle sprain has been explored by few researchers [5, 10, 14, 30, 39]. Both acute and chronic ankle instability were successfully developed in the mouse model by performing ligamentectomy. Apart from the surgical method, a manually induced ankle sprain model was attempted by Kim et al. [22], which was eventually proved to have difficulty in specifying the injured ligament. In addition, the manual method may cause damage to surrounding structures, such as muscles and tendons, which might confound the data. Therefore, surgical modeling is the most suitable method for such experiments. Pain is an inevitable complication of surgical modeling. According to our observations and the results of *Wikstrom* et al., the pain disappeared in about 3 days and proved to have no significant effect on the experimental results [39]. The initiation of ankle OA is usually featured by cartilage degeneration, which was observed in the mouse experiments. The ankle joint is more resistant to cartilage degeneration since it is subjected to the greatest mechanical loading among all joints. Thus, wheel training was applied postoperatively on mice to accelerate the development of ankle OA, which has been proved repeatable and stable in mice by Knab et al. [24]. In conclusion, such a surgically-induced animal model of ankle sprain can promote our understanding of the ankle OA mechanism.

### Comparison between mouse and human ankle joints

It is known that mice have rather high genetic similarities with humans, thus it is reasonable to exploit it in different kinds of studies. Mice and humans are also highly similar when it comes to foot and ankle studies. The bone components and ligament location in the mouse ankle are exactly the samein that of humans [22]. More specifically, the stability of lateral ankle in the mice is maintained by the ATFL, PTFL, and CFL, while the deltoid ligament (DTL) is basically responsible for stabilizing the medial ankle, which is almost identical in humans. In contrast, the ATFL in mice plays a less important role in maintaining the stability of the lateral ankle in mice than in humans. In our previous study, the muscles, bones, and cartilage of mouse and human ankle were compared using micro-CT, and it was found that the mouse and human ankle joint were very similar, with only exception that the talar dome of the mouse appeared more symmetrical compared to that of human, and it had a groove on it. Moreover, similar skeletal properties in the mouse and human talocrural joints were observed, including malleolar width and thickness and trochlea tali arc length and width. Based on the similarities between mice and human ankles, mice are currently considered to be one of the most suitable experimental animals for simulating ankle sprains.

### Behavior test analysis

The results of the balance beam experiment showed that at 8 weeks after operation, the average time for the mice of the three groups to pass the balance beam increased compared to that before the operation. This may be related to the ankle instability after ankle sprain and the fear of walking on uneven roads, which can lead to an increase in the passage time. No significant difference was observed between the CFL group and the SHAM group in the acute phase of injury within 2 weeks after surgery. However, the balance ability of the CFL + ATFL group began to decline significantly. At 8 weeks after operation, the number of slips of the mice in the CFL + ATFL group was higher than that in the CFL group and the SHAM group, which indicated that the more severe the ankle injury, the more obvious the change in the balance ability of the mice. These results suggested that mice with less ligament damage can recover mobility more quickly, which is consistent with previous studies [23, 28, 33, 40]. Meanwhile, the results on balance outcomes were similar to those in existing literature. The number of slips observed during the balance tests were similar to those reported by Carter *et al.* [6] The results of balance ability can reflect the progress of ankle joint instability and ankle OA in mice, however this often needs to be combined with footprint analysis. Compared to the research by Turner et al., all groups in the present study had shorter time to pass the balance beam and slipped less times, possibly due to that ankle instability was not as severe as in their study [20]. Turn-around and pole tests are also common methods that have been used as behavior tests. However, they were not suitable for the present study, since they are mostly used in assessing behavior changes caused by neurological diseases.

### Hints from gait analysis

Classical gait analysis of human motion includes step length, stance length, stride length, and stride width. Mice have long been used to simulate human gait. In mice, the paw overlap should also be considered as a parameter during gait analysis, which is different from humans. CatWalk is a video-based automated gait analysis system that has been widely used in rodents [1, 3, 12].In addition, the Digi Gait Imaging System has been used in mice with arthritis [36]. In the current study, both the static and the dynamic phase of gait were measured. Previous studies have rarely involved gait analysis, but we think gait analysis is also very convincing although there will be inevitable individual differences [19, 20].

It is worth noting that in the CFL and SHAM groups, both the fore and hind stride length increased gradually after operation and eventually decreased to less than 1, while in the CFL + ATFL group it basically remained above 1 (Fig. 3A and B). More specifically, the mice in the CFL + ATFL group were reluctant to use their left ankle, thus their right stride length was always longer than the left. The results of the CFL and the SHAM groups were close and lower than 1, which may be due to the relatively mild ligament damage in the CFL group, where the ligament began to repair itself about 3 weeks after surgery. The foot base width reflects the relationship between the inner and outer ankle muscle strength during walking. Since only the hind feet of the mice were modeled in the experiment, the width of the forefoot was significantly smaller than that of the hind foot, which can be clearly observed in Fig. 3F and G. Due to that the lateral ligaments of the ankle were destroyed, the mice had to narrow the distance between their feet in order to reduce the pressure of their weight on the ankle. Therefore, whether in the static or dynamic phase, the distance in the experimental group was smaller than that in the control group (Fig. 3E, F, and G).The present results were similar with those of Erik et al., however the training time in that study was longer, and the final conclusion was consistent [39]. The gait analysis results indicated that in mice with severe ankle sprain, the ankle joint stability was worse and the walking function was further decreased. Although physical activity data remain relatively sparse in the human CAI literature, the results of the present study coincide with available empirical data [18, 35].

### Joint degeneration revealed by histology

Histological staining is a highly reliable method, which is commonly used to evaluate OA severity in animal models. Among all staining methods, Saffranin-O/Fast green is one of the most commonly used, since it can clearly distinguish cartilage from bone tissue and can accurately and intuitively assess the degree of ankle OA. One of the advantages of a mouse model is the small joint size, which enables the collection of sections encompassing the entire joint. In this study, the OARSI score indicated that there was basically no significant ankle joint degeneration in the SHAM group, moderate degree of osteoarthritis was found in the CFL group, while significant joint degeneration was found in the CFL + ATFL group. The histological analysis results were consistent with those of the gait analysis, suggesting that the severe ankle instability (CFL + ATFL) group presented more obvious OA signs than the moderate ankle instability (CFL) group. Analyzing the above results, it is not difficult to find that in mice, the more severe the injury of the lateral ligament of the ankle joint, the worse the stability of the ankle joint and the higher the incidence of OA, which applies also to humans. Previous studies have not basically involved the probability of severe ankle instability leading to ankle osteoarthritis. The histological analysis results in the current work were consistent with those of Chang *et al.* [7] However, our study further concluded that severe ankle instability is three times more likely than moderate ankle instability to lead to ankle osteoarthritis, which makes the understanding of the disease more quantitative and intuitive.

### Correlation with clinical significance

Clinical ankle instability is not uncommon, but due to the lack of understanding of its pathogenesis, most people fail to receive proper treatment. Eventually, the pathogenesis develops into ankle joint OA and the patients have to undergo joint replacement or fusion surgery [2, 27].This study conducted behavioral tests, gait analysis, and histological analysis on mice, in order to fully explore the relationship between different ligament injuries and ankle instability, which provided us with a new understanding of the importance of CFL,ATFL, and other ligaments.

This study was conducted in mice and the conclusions should be carefully extended to humans as there are some differences between mice and humans. Based on the conclusion of this study, we are inspired that there may also be serious CFL and ATFL ligament injuries in human ankle joint that lead to ankle instability, and then accelerate the process of joint degeneration, which needs further and in-depth research. This present study may promote the development of ligamentous injury ankle instability research and offer new concepts in translational orthopedics research of instability in the ankle joint.

In practical clinical application, it is necessary to carefully examine the ankle function of the patient [29], since different ligament injuries can cause varying degrees of ankle joint instability. The more the number of damaged ligaments, the more severe the injury and the higher the risk of developing ankle OA. Therefore, physicians should try to restore the ligament function of patients by performing ligament repair surgery or applying a brace, in order to delay the progress of the disease. This study can provide some guidance for clinical work.

## Limitations

This study focused on the biomechanics of ankle OA and committed to transforming the results into clinical practice. However, the treatment countermeasure was not involved and will be included in our future studies. On the other hand, the number of experimental animals used in this study was very small; thus, in future studies, the number of samples will be appropriately increased in order to get more sufficient data. The relationship between ankle instability and degeneration of adjacent joints, such as the subtalar joint and the talonavicular joint, will also be investigated in our future work. Another limitation of this study is that, due to the limitations of the animal model, the sensorimotor factors could not be taken into account. Further experiments will be performed in our corresponding research in the future. Since the main purpose of this experiment is to test whether there is any difference between the model before and after, the comparison of time before and after each group is only carried out, and the comparison between groups is not involved, which will be improved in the subsequent experiment.

## Conclusion

The present study demonstrated that the group of mice with more severe ankle ligament injury (CFL + ATFL) was less stable than that with moderate ankle injury (CFL) and had almost three times higher possibility to develop PTOA, which has significant implication for early clinical diagnosis and ankle OA-related disease prevention.

## Supplementary Information

**Additional file 1: Appendix 1**

**Additional file 2: Appendix 2**

**Additional file 3: Appendix 3**

## Data Availability

The datasets generated and analyzed during the current study are not publicly available due to limitations of ethical approval involving the patient data and anonymity but are available from the corresponding author on reasonable request.
