# Supplementary material for: Ligamentous injury-induced ankle instability causing posttraumatic osteoarthritis in a mouse model
Source: BMC Musculoskelet Disord. 2022 Mar 8;23:223. doi: 10.1186/s12891-022-05164-5 (PMC8905815; doi:10.1186/s12891-022-05164-5)
Supplement: Supplementary file 1 — Additional file 1: Appendix 1 [file 12891_2022_5164_MOESM1_ESM.docx]

Statistical analysis was performed using the SPSS statistical package. Two-way ANOVA (group x time) was used to cross-examine the means of the data among three groups at the same point of time. Paired Student's t-test was used for each group along the time axis to examine if the data has increased/decreased as the study processed. A p-value <0.05 was considered statistically significant.

**Fore Stride Length Asymmetry**

|  | **CFLvsSHAM** | **CFL+ATFLvsSHAM** | **CFLvsCFL+ATFL** |
| --- | --- | --- | --- |
| **DO** | 0.4104460 | 0.35920447 | 0.2149627 |
| **D3** | 0.5947008 | 0.58396078 | 0.9758390 |
| **1W** | 0.5913514 | 0.69815609 | 0.9304572 |
| **2W** | 0.3157886 | 0.39588012 | 0.7831210 |
| **3W** | 0.4642311 | 0.14147966 | 0.4507619 |
| **4W** | 0.7229962 | 0.08280725 | 0.2039680 |
| **5W** | 0.3402436 | 0.96763028 | 0.3521412 |
| **7W** | 0.9520530 | 0.10629148 | 0.2236285 |

There is no significant difference among different groups.

|  | **SHAM** | **CFL** | **CFL+ATFL** |
| --- | --- | --- | --- |
| **D3vsD0** | 0.1727251 | 0.17553305 | 0.23762808 |
| **1WvsD0** | 0.3931703 | 0.05989391 | 0.30310327 |
| **2WvsD0** | 0.3760155 | 0.04623411 | 0.20144637 |
| **3WvsD0** | 0.1424735 | 0.37384738 | 0.19983083 |
| **4WvsD0** | 0.1351467 | 0.48446430 | 0.20500034 |
| **5WvsD0** | 0.4412359 | 0.37937534 | 0.15486987 |
| **7WvsD0** | 0.4465974 | 0.15930277 | 0.09188799 |

SHAM and CFL+ATFL group shows no significant change through this study. CFL group shows significant increase in the first week (p=0.046) and then no significant change after that.

**Hind Stride Length Asymmetry**

|  | **CFLvsSHAM** | **CFL+ATFLvsSHAM** | **CFLvsCFL+ATFL** |
| --- | --- | --- | --- |
| **DO** | 0.8313307 | 0.59554700 | 0.78250085 |
| **D3** | 0.1064684 | 0.55537489 | 0.32375176 |
| **1W** | 0.9186693 | 0.95422608 | 0.88503575 |
| **2W** | 0.3273736 | 0.63896500 | 0.65708091 |
| **3W** | 0.6551576 | 0.36337146 | 0.59682852 |
| **4W** | 0.8038859 | 0.11195864 | 0.22562857 |
| **5W** | 0.1050744 | 0.66115619 | 0.08071573 |
| **7W** | 0.5832651 | 0.05994835 | 0.23653282 |

There is no significant difference among different groups.

|  | **SHAM** | **CFL** | **CFL+ATFL** |
| --- | --- | --- | --- |
| **D3vsD0** | 0.21369565 | 0.4852914 | 0.40279382 |
| **1WvsD0** | 0.39818880 | 0.4069720 | 0.47679629 |
| **2WvsD0** | 0.12200296 | 0.4355948 | 0.07530289 |
| **3WvsD0** | 0.14005343 | 0.4656010 | 0.09435247 |
| **4WvsD0** | 0.05151789 | 0.4946774 | 0.17148174 |
| **5WvsD0** | 0.12043899 | 0.4543143 | 0.09857952 |
| **7WvsD0** | 0.22027367 | 0.4707702 | 0.10123216 |

There is no significant difference among different groups.

**Overlap Symmetry**

|  | **CFLvsSHAM** | **CFL+ATFLvsSHAM** | **CFLvsCFL+ATFL** |
| --- | --- | --- | --- |
| **DO** | 0.06887444 | 0.9606235327 | 0.100411889 |
| **D3** | 0.34499863 | 0.4762975684 | 0.089295867 |
| **1W** | 0.02643220 | 0.0809497499 | 0.087616637 |
| **2W** | 0.34393369 | 0.2531142294 | 0.332477615 |
| **3W** | 0.19819131 | 0.6951136449 | 0.142170265 |
| **4W** | 0.92278931 | 0.2043445044 | 0.155154241 |
| **5W** | 0.23866155 | 0.6841861218 | 0.196407509 |
| **7W** | 0.69098698 | 0.0009442802 | 0.002052079 |

At the first week, CFL group shows significant difference from SHAM group (p = 0.026) and CFL+ATFL group shows difference from SHAM group (p<0.1). CFL+ATFL shows significant difference from other two groups in the 7^th^ week (p<0.05).

|  | **SHAM** | **CFL** | **CFL+ATFL** |
| --- | --- | --- | --- |
| **D3vsD0** | 0.10334678 | 0.05081798 | 0.2086690010 |
| **1WvsD0** | 0.33294756 | 0.06723927 | 0.0957320693 |
| **2WvsD0** | 0.06215651 | 0.16799869 | 0.3562644281 |
| **3WvsD0** | 0.02993271 | 0.06496903 | 0.0718837355 |
| **4WvsD0** | 0.03477365 | 0.08134369 | 0.0570512139 |
| **5WvsD0** | 0.01213854 | 0.08917774 | 0.0875616296 |
| **7WvsD0** | 0.07755180 | 0.38915630 | 0.0005164317 |

SHAM and CFL group show no significant change along the time axis. CFL+ATFL group has a significant decrease in the last week (p<0.05).

**Fore Stance Length**

|  | **CFLvsSHAM** | **CFL+ATFLvsSHAM** | **CFLvsCFL+ATFL** |
| --- | --- | --- | --- |
| **DO** | 0.4792764 | 0.09990505 | 0.4659916 |
| **D3** | 0.8123135 | 0.86892402 | 0.9213992 |
| **1W** | 0.9366668 | 0.34827169 | 0.3090164 |
| **2W** | 0.1298043 | 0.02100047 | 0.2031796 |
| **3W** | 0.6964496 | 0.25165622 | 0.1668371 |
| **4W** | 0.1395705 | 0.88301508 | 0.1715593 |
| **5W** | 0.9672478 | 0.17869654 | 0.1233058 |
| **7W** | 0.2459598 | 0.62603062 | 0.4595348 |

CFL+ATFL group shows significant difference from SHAM group in the second week. There is no other significant difference.

|  | **SHAM** | **CFL** | **CFL+ATFL** |
| --- | --- | --- | --- |
| **D3vsD0** | 0.135746078 | 0.36214321 | 0.373930109 |
| **1WvsD0** | 0.263931423 | 0.44504603 | 0.300757195 |
| **2WvsD0** | 0.064394784 | 0.02386605 | 0.001516937 |
| **3WvsD0** | 0.314587442 | 0.19391257 | 0.070441944 |
| **4WvsD0** | 0.002027401 | 0.03498662 | 0.009933623 |
| **5WvsD0** | 0.010263965 | 0.02536763 | 0.001269053 |
| **7WvsD0** | 0.002014747 | 0.02578250 | 0.008827233 |

In the second week, CFL and CFL+AFL group has a significant increase(p<0.05) and SHAM group has a increase (p<0.1). All groups show significant increase from the 4^th^ week to the last week (p<0.05).

**Hind Stance Length**

|  | **CFLvsSHAM** | **CFL+ATFLvsSHAM** | **CFLvsCFL+ATFL** |
| --- | --- | --- | --- |
| **DO** | 0.81973186 | 0.1375339 | 0.21190990 |
| **D3** | 0.42195054 | 0.6969774 | 0.64320418 |
| **1W** | 0.90908184 | 0.1740493 | 0.16510410 |
| **2W** | 0.87201924 | 0.5114092 | 0.52994489 |
| **3W** | 0.85127139 | 0.1610878 | 0.04317894 |
| **4W** | 0.04173011 | 0.4004188 | 0.12911658 |
| **5W** | 0.79996759 | 0.1753680 | 0.27218034 |
| **7W** | 0.06685383 | 0.2395314 | 0.30091414 |

CFL group shows significant difference from SHAM group in the 4th week and 7th week. CFL+ATFL shows significant difference from CFL group in the third week.

|  | **SHAM** | **CFL** | **CFL+ATFL** |
| --- | --- | --- | --- |
| **D3vsD0** | 0.046044426 | 0.126475816 | 0.370809876 |
| **1WvsD0** | 0.083211142 | 0.159108550 | 0.049126829 |
| **2WvsD0** | 0.005897921 | 0.033645491 | 0.006913137 |
| **3WvsD0** | 0.086406926 | 0.010686460 | 0.004471650 |
| **4WvsD0** | 0.001337287 | 0.013802951 | 0.007584109 |
| **5WvsD0** | 0.007218526 | 0.006498686 | 0.000959175 |
| **7WvsD0** | 0.002630640 | 0.021487433 | 0.015075201 |

SHAM group shows great instability throughout this study. It shows significant difference in D3, 2W, 4W, 5W, and 7W(p<0.05). Both CFL and CFL+ATFL group shows significant increase starting from the second week to the last week (p<0.05).

**Fore Base Width**

|  | **CFLvsSHAM** | **CFL+ATFLvsSHAM** | **CFLvsCFL+ATFL** |
| --- | --- | --- | --- |
| **DO** | 0.9552451 | 0.66965629 | 0.6369413 |
| **D3** | 0.6135175 | 0.10267620 | 0.3429327 |
| **1W** | 0.0758607 | 0.56185174 | 0.1533609 |
| **2W** | 0.8445044 | 0.75457685 | 0.8976661 |
| **3W** | 0.1874226 | 0.09651097 | 1.0000000 |
| **4W** | 0.4522077 | 0.69085486 | 0.7637824 |
| **5W** | 0.3383937 | 0.76896262 | 0.5467447 |
| **7W** | 0.3014762 | 0.04981209 | 0.4882673 |

There is no significant difference among different groups.

|  | **SHAM** | **CFL** | **CFL+ATFL** |
| --- | --- | --- | --- |
| **D3vsD0** | 0.3169117 | 0.49380272 | 0.3076184 |
| **1WvsD0** | 0.2661475 | 0.08656977 | 0.3729076 |
| **2WvsD0** | 0.2902428 | 0.25214413 | 0.2039752 |
| **3WvsD0** | 0.2531266 | 0.21030974 | 0.3529662 |
| **4WvsD0** | 0.4160631 | 0.27562364 | 0.3867462 |
| **5WvsD0** | 0.2425705 | 0.39073330 | 0.2208370 |
| **7WvsD0** | 0.1401854 | 0.50000000 | 0.1435071 |

There is no significant change of each group along the time axis.

**Hind Base Width**

|  | **CFLvsSHAM** | **CFL+ATFLvsSHAM** | **CFLvsCFL+ATFL** |
| --- | --- | --- | --- |
| **DO** | 0.44787774 | 0.4516496 | 0.84744714 |
| **D3** | 0.61765511 | 0.9108361 | 0.58208198 |
| **1W** | 0.89265511 | 0.2748802 | 0.33957451 |
| **2W** | 0.06092196 | 0.1116414 | 0.49582564 |
| **3W** | 0.01768226 | 0.3401320 | 0.03272865 |
| **4W** | 0.01180053 | 0.1216299 | 0.20276217 |
| **5W** | 0.09587857 | 0.1015785 | 0.70221181 |
| **7W** | 0.23714554 | 0.8906646 | 0.16821753 |

CFL group shows significant difference from SHAM group in the 3rd week and 4th week (p<0.05) and difference in the 2nd week and 5th week (p<0.1). CFL+ATFL group shows significant difference from CFL group in the 3rd week (p<0.05).

|  | **SHAM** | **CFL** | **CFL+ATFL** |
| --- | --- | --- | --- |
| **D3vsD0** | 0.409746218 | 0.08743711 | 0.24618666 |
| **1WvsD0** | 0.407477669 | 0.17305846 | 0.05458561 |
| **2WvsD0** | 0.040222653 | 0.39814854 | 0.18148792 |
| **3WvsD0** | 0.004869188 | 0.07425350 | 0.01104889 |
| **4WvsD0** | 0.004273952 | 0.26195997 | 0.05806882 |
| **5WvsD0** | 0.002326953 | 0.02576928 | 0.01812072 |
| **7WvsD0** | 0.037185660 | 0.11993025 | 0.01676459 |

SHAM group shows significant increase hindbase width starting from the 3rd week to the last week (p<0.05). In the contrast, CFL group did not show any significant increase until the 5th week (p<0.05). CFL+ATFL group show significant increase on 3rd week, 5th week and 7th week (p<0.05).
