# Supplementary figures and images for: Ligamentous injury-induced ankle instability causing posttraumatic osteoarthritis in a mouse model
Source: BMC Musculoskelet Disord. 2022 Mar 8;23:223. doi: 10.1186/s12891-022-05164-5 (PMC8905815; doi:10.1186/s12891-022-05164-5)

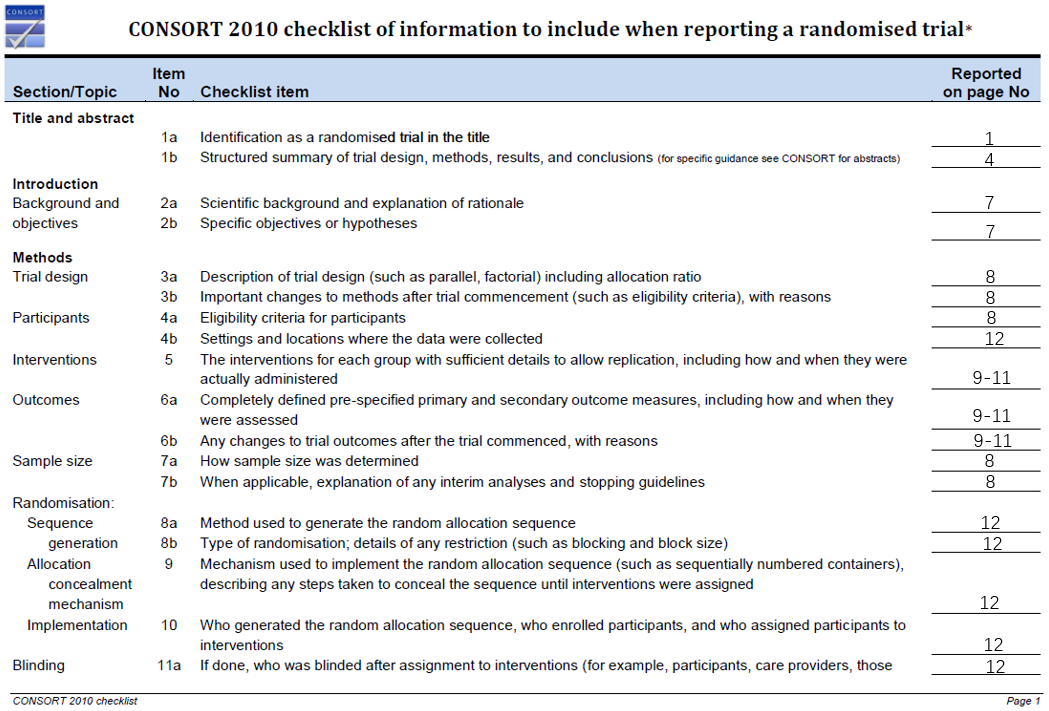

Supplement: Supplementary file 2 — Additional file 2: Appendix 2 [file 12891_2022_5164_MOESM2_ESM.png]

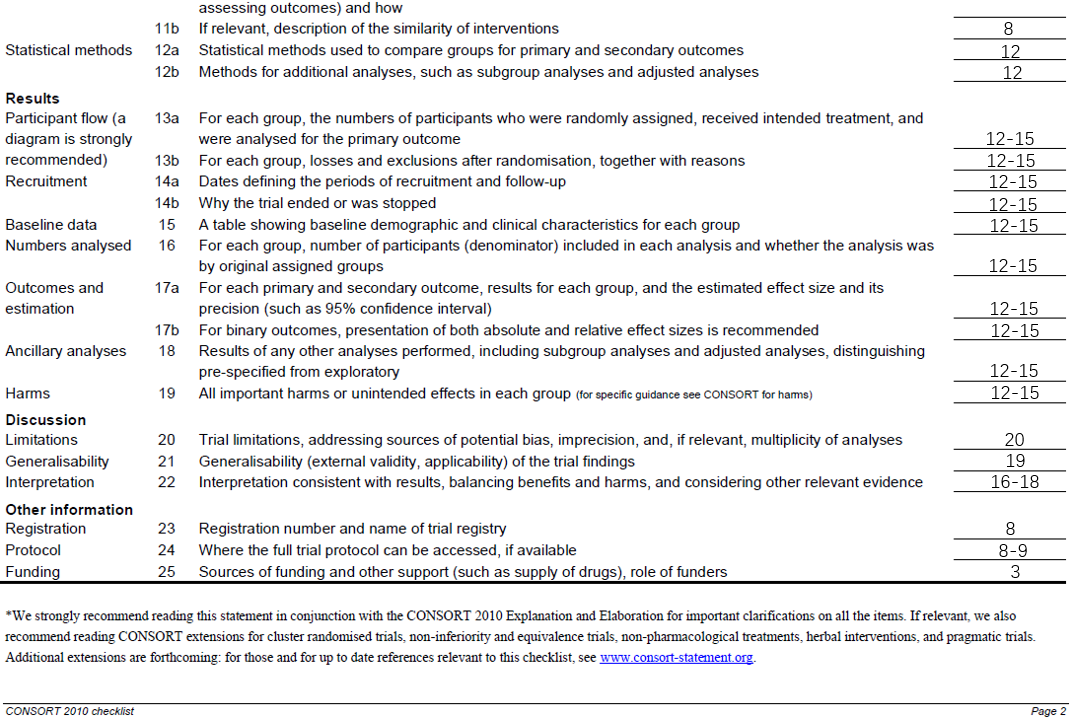

Supplement: Supplementary file 3 — Additional file 3: Appendix 3 [file 12891_2022_5164_MOESM3_ESM.png]
